# Selective inhibition of JNK mitochondrial location is protective against seawater inhalation-induced ALI/ARDS

**DOI:** 10.3892/mmr.2021.12154

**Published:** 2021-05-14

**Authors:** Liyan Bo, Yanyan Li, Wei Liu, Faguang Jin, Congcong Li

**Affiliations:** 1 Department of Respiratory and Critical Care Medicine, General Hospital of Northern Theater Command, Shenyang, Liaoning 110016, P.R. China; 2 Department of Respiratory and Critical Care Medicine, Tangdu Hospital, Fourth Military Medical University, Xi'an, Shaanxi 710038, P.R. China; 3 Department of Respiratory and Critical Care Medicine, Chest Hospital of Xi'an, Xi'an, Shaanxi 710038, P.R. China

**Keywords:** JNK, acute lung injury and acute respiratory distress syndrome, mitochondria, apoptosis, autophagy

## Abstract

Localization of phosphorylated (p)-JNK to the mitochondria can lead to functional mitochondrial disorder, resulting in a decrease in energy supply and membrane potential, as well as an increase in reactive oxygen species production and apoptosis. JNK is involved in the occurrence of acute lung injury (ALI), and activation of the JNK pathway is one of the crucial factors resulting in injury. The aim of the present study was to investigate whether the JNK-mitochondria (mitoJNK) location participated in the occurrence of ALI and acute respiratory distress syndrome (ALI/ARDS). The present study examined the activation of the JNK pathway, the content of JNK located on the mitochondria and the treatment effects of a cell-permeable peptide Tat-Sab_KIM1_, which can selectively inhibit the location of JNK on mitochondria. The expression levels of proteins were detected by western blot analysis. Lung injuries were evaluated by histological examination, wet-to-dry weight ratios, and H_2_O_2_ and malondialdehyde concentrations in the lung tissues. Lung cells apoptosis was evaluated using TUNEL assay. The results demonstrated that JNK was phosphorylated and activated during seawater inhalation-induced ALI/ARDS, not only in the routine JNK pathway but also in the mitoJNK pathway. It was also found that Tat-Sab_KIM1_ could specifically inhibit JNK localization to mitochondria and the activation of mitoJNK signaling. Furthermore, Tat-Sab_KIM1_ could inhibit Bcl-2-regulated autophagy and mitochondria-mediated apoptosis. In conclusion, mitoJNK localization disrupted the normal physiological functions of the mitochondria during ALI/ARDS, and selective inhibition of JNK and mitochondrial SH3BP5 (also known as Sab) binding with Tat-Sab_KIM1_ can block deterioration from ALI/ARDS.

## Introduction

JNK belongs to the family of MAPKs and encompasses three encoded genes, *JNK1, JNK2* and *JNK3* (1). *JNK1* and *JNK2* are widely expressed in various tissues, whereas *JNK3* is primarily expressed in the brain and heart (1,2). JNK is also known as stress-activated kinase, which is involved in the regulation of the response of the body to external or internal stimuli, inflammation and cell apoptosis (3). As a response to a specific stimulation, MAPKs [such as MAPK kinase (MKK) 4 and MKK7] activate JNK via phosphorylation (4), and subsequently, regulate the phosphorylation and activity of several downstream factors, such as activating transcription factor-2 (5), ETS transcription factor (6) and nuclear factor of activated T-cells (7). Among these factors, c-jun/activator protein-1 (AP-1) has been clearly elucidated with respect to the JNK regulatory pathway. JNK can activate AP-1 via phosphorylation, followed by the regulation of cell proliferation and apoptosis (8). Several pathological studies (9,10) have reported the abnormal activation of the JNK signaling pathway and its involvement in the modulation of cell apoptosis, and inhibition of this pathway can reduce the proportion of apoptotic cells under pathological conditions (9). Furthermore, JNK can be activated via phosphorylation, and bind to the outer membrane mitochondrial protein mitochondrial SH3BP5 (also known as Sab), thereby regulating the function of this organelle (11). The localization of p-JNK to mitochondria can lead to its functional disorder, resulting in a decrease in the energy supply and membrane potential, as well as an increase in reactive oxygen species (ROS) production and the occurrence of apoptosis (8,9,12).

Previous studies (13–15) have shown that JNK was involved in the occurrence of acute lung injury (ALI) and served an important role in the regulation of apoptosis in lung tissue. Experimental studies have revealed that the activation of the JNK pathway was one of the crucial factors resulting in injury; this pathway also interacts with the NF-κB pathway, intercellular tight junction proteins and TGF-β to participate in the development of lung injury (13,16). The pathological changes associated with ALI are partly due to the abnormal regulation of mitochondria, such as the production of ROS, induced autophagy and apoptosis (17–19). Moreover, maintaining the stability of mitochondrial function is vital to ameliorate ALI/acute respiratory distress syndrome (ARDS) (20,21). Mitochondria, as intracellular energy sources, serve a crucial role in cell survival and the normal physiological function of organelles, and maintaining mitochondrial stability is essential for cells, tissues and organs (22). Activation of the JNK pathway has been reported to disrupt the function of mitochondria in myocardial ischemia-reperfusion injury, and as a result, decreases the function and survival of myocardial cells (8). However, activation of JNK-mediated mitochondrial function abnormalities is rarely reported with respect to the occurrence and progression of ALI/ARDS. In the present study, we hypothesized that abnormal activation of the JNK-mitochondrial (mitoJNK) pathway could significantly disrupt the normal physiological function of lung cells, resulting in the occurrence of ALI/ARDS. Moreover, the underlying role of the mitoJNK pathway in ALI remains unknown, and requires further study.

## Materials and methods

### 

#### Reagents

Antibodies (Ab) against cytochrome c (cat. no. ab133504), Bcl-2 (cat. no. ab196495), cytochrome c oxidase IV (COX IV) (cat. no. ab202554) and GAPDH (cat. no. ab181602) were purchased from Abcam. Phosphorylated (p)-Bcl-2 (Ser70) rabbit monoclonal (m)Ab (cat. no. #2827), p-JNK (Thr183/Tyr185) rabbit mAb (cat. no. #4668) and JNK rabbit mAb (cat. no. #9258) were obtained from Cell Signaling Technology, Inc. LC3 rabbit mAb (cat. no. sc-398822) was purchased from Santa Cruz Biotechnology, Inc. The malondialdehyde (MDA) Assay kit (TBA method) (cat. no. A003-1) and H_2_O_2_ Assay kit (cat. no. A064-1) were obtained from Nanjing Jiancheng Bioengineering Institute. An *In Situ* Cell Death Detection kit was obtained from Roche Diagnostics GmbH. Dexamethasone (DXM) was purchased from Sigma-Aldrich (Merck KGaA). Tat-Sab_KIM1_ (GFE SLS VPS PLD LSG PRV VAPPRRRQRRKKRG-NH_2_) was purchased from NeoPeptide. Seawater (osmolality 1,300 mmol/l; pH 8.2; 26.518 g/l NaCl; 3.305 g/l MgSO_4_; 2.447 g/l MgCl_2_; 1.141 g/l CaCl_2_; 0.725 g/l KCl; 0.202 g/l NaHCO_3_; 0.083 g/l NaBr) was prepared according to the major composition of the East China Sea provided by the Chinese Ocean Bureau (23).

#### Animal procedures

The animal procedures in this study were approved by the Animal Care and Use Committee of The Fourth Military Medical University (approval no. TDLL20160193), and were carried out in accordance with the Declaration of the National Institutes of Health Guide for Care and Use of Laboratory Animals (24). A total of 32 male Sprague-Dawley (SD) rats (weight, 180–220 g; age, 6 weeks) were maintained on a light/dark cycle of 12:12-h with free access to food and water. The rats were maintained in an atmosphere with an ambient temperature of 18–26°C and relative humidity of 40–70%. The SD rats were randomly divided into the following experimental groups (n=8): i) Control group; ii) seawater inhalation groups; iii) DXM pre-treatment group, in which the rats were pre-treated intraperitoneally with DXM (2.5 mg/kg body weight) 30 min before modelling; and iv) Tat-Sab_KIM1_ pre-treatment group, in which the rats were pre-treated with Tat-Sab_KIM1_ (2 mg/kg body weight) via tracheal injection 10 min before modelling.

Seawater inhalation-induced ALI/ARDS was established through the following procedures. First, the rats were anesthetized with sodium pentobarbital (45 mg/kg weight) intraperitoneally. Then, the animals were placed in the supine position with the head elevated at an angle of 30° during the experiments. A 1-ml syringe was gently inserted into the trachea at ~1.5 cm above the carina. Subsequently, 4 ml/kg body weight seawater was instilled into both lungs within 4 min. The lungs were then harvested after the rats were sacrificed via intraperitoneal injection of 200 mg/kg sodium pentobarbital at the predetermined time (6 h).

#### Western blot analysis

The protein extract prepared from the lung tissue harvested 6 h after seawater instillation was subjected to western blot analysis, as described previously (25). Briefly, the protein was obtained using RIPA lysis buffer (Beyotime Institute of Biotechnology) and protein concentration was quantified using a Bradford kit (Beyotime Institute of Biotechnology) according to the manufacturer's instructions. Total proteins (20 µg) were separated by SDS-PAGE on 12% gels and transferred to nitrocellulose membranes (EMD Millipore). Then, the membranes were blocked with 10% non-fat dry milk in TBS at room temperature for 30 min and probed at 4°C overnight with primary Abs, including anti-p-JNK (1:1,000), anti-JNK (1:1,000), anti-GAPDH (1:2,500), anti-COX IV (1:1,000), anti-LC3 (1:1,000), anti-p-Bcl-2 (1:1,000), anti-Bcl-2 (1:1,000) or anti-cytochrome c (1:10,000). Subsequently, the membranes were washed with TBS-0.3%Tween-20 and then incubated with an appropriate HRP-conjugated secondary Ab (1:10,000) at room temperature for 2 h. The immunoreactive target proteins were detected using an ECL detection system (Thermo Fisher Scientific, Inc.). Band intensities were semi-quantified using Image Lab 4.1 (Bio-Rad Laboratories, Inc.).

#### Histopathological evaluation

The lung tissue was harvested and fixed with 4% paraformaldehyde at room temperature for 24 h, embedded in paraffin and cut into 5-µm sections that were stained with H&E at room temperature for 3 min. Sections were examined with an optical microscope (magnification, ×100).

#### Preparation of mitochondrial and cytosolic/nuclear proteins

Mitochondrial and cytosolic/nuclear proteins were prepared by isolating mitochondria from the cells, as described previously (9). Briefly, isolation buffer (210 mM mannitol; 70 mM sucrose; 5 mM HEPES; 1 mM EGTA; 0.5 mg/ml BSA (Merck KGaA; pH=7.4) was used to wash and homogenize the rat lungs. Then, the homogenate was centrifuged at 1,000 × g for 10 min at 4°C. The supernatant was collected and centrifuged at 10,000 × g for 10 min at 4°C. This second supernatant was used as the soluble cytosolic/nuclear fraction with excluded mitochondria, and the sedimentation pellet was resuspended in lysis buffer for western blot analysis of the mitochondrial proteins according to the aforementioned steps. COX IV was used as an internal mitochondrial control, and GAPDH served as the control for other organelles.

#### Lung wet-to-dry (W/D) weight ratio

The W/D weight ratio of the lung tissue is commonly used to reflect the severity of pulmonary oedema (25). Briefly, the rats were sacrificed at the predetermined time points (6 h after seawater inhalation), and the chests were quickly opened. The same part of the left lung from each rat was weighed as the wet weight after removal of the other tissues. Then, each lung was placed in an oven for baking at 80°C for 72 h to obtain a constant weight, denoted as the dry weight. The W/D weight ratio was obtained by dividing the wet weight by the dry weight of the left lung.

#### Determination of H_2_O_2_ concentrations and MDA levels

H_2_O_2_ and MDA concentrations in the lung tissues were detected using the H_2_O_2_ Assay kit and MDA Assay kit, respectively, according to the manufacturer's instructions. Briefly, the lung tissue samples were homogenized in cold normal saline (lung tissue to normal saline ratio, 1:9). Then, the homogenate was examined according to the protocol of the kit. To detect the H_2_O_2_ concentration, the rate of change in absorbance was measured with a spectrophotometer at 405 nm. For detecting the MDA concentration, the rate of change in absorbance was measured with a spectrophotometer at 532 nm.

#### Assessment of lung cells apoptosis

In order to quantify cell apoptosis in the injured rat lungs, a TUNEL assay was conducted using an *In Situ* Cell Death Detection kit according to the manufacturer's instructions. Briefly, lung tissues were fixed with 4% paraformaldehyde for 24 h at room temperature. The lung tissues were then embedded in paraffin and sectioned into 5-µm sections. After dewaxing, the tissue sections were incubated with TUNEL working solution for 1 h at 37°C to label apoptotic cells, and then nuclei were stained with DAPI (Merck KGaA) for 5 min at room temperature. The slides were mounted with 50% glycerol (Merck KGaA). Finally, the sections were visualized using a fluorescence microscope (magnification, ×200; Olympus Corporation). A total of 10 images from three slides from every group were randomly selected, and the number of cells exhibiting positive staining for apoptosis per field were counted and analysed.

#### Statistical analysis

Statistical analyses were performed using GraphPad Prism 8 (GraphPad Software, Inc.). The graphical data are presented as the mean ± SEM and experiments were repeated four times. The experimental groups were compared using a one-way ANOVA and Bonferroni's multiple comparison tests. P<0.05 was considered to indicate a statistically significant difference.

## Results

### 

#### mitoJNK signaling is activated during seawater inhalation-induced ALI/ARDS

**Figure 1.**
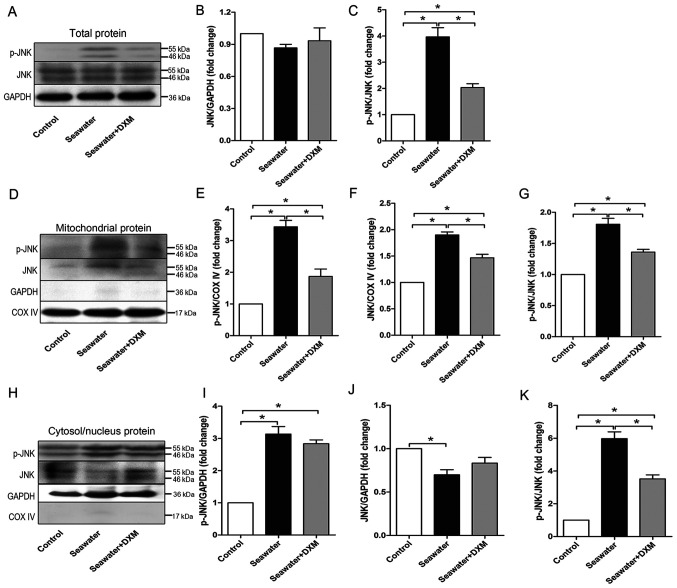
JNK-mitochondria signaling is activated during seawater inhalation-induced acute lung injury/acute respiratory distress syndrome. (A) Representative western blotting images of p-JNK and JNK expression in the total protein. (B) Relative JNK protein content in the total protein. (C) Normalized ratio of p-JNK/JNK of the total protein. (D) Representative western blotting images of p-JNK and JNK expression in the mitochondrial protein. Relative (E) p-JNK and (F) JNK protein content in the mitochondrial protein. (G) Normalized ratio of p-JNK/JNK in the mitochondrial protein. (H) Representative western blotting images of p-JNK and JNK expression in the cytosol/nucleus protein. Relative (I) p-JNK and (J) JNK protein content in the cytosol/nucleus protein. (K) Normalized ratio of p-JNK/JNK of the cytosol/nucleus protein. Data are presented as the mean ± SEM. n=4/group. *P<0.05. DXM, dexamethasone; p-, phosphorylated; COX IV, cytochrome c oxidase IV.

To elucidate the role of JNK, especially mitoJNK, in ALI/ARDS, the current study assessed the expression levels of p-JNK and JNK in the total protein, mitochondrial protein and cytosol/nucleus protein of the lung via western blot analysis. As shown in Fig. 1A, B and C, seawater inhalation significantly phosphorylated JNK, thereby activating the JNK pathway in the lung. Furthermore, it was found that the phosphorylation level of JNK was elevated significantly in both the mitochondria (Fig. 1D, E and G) and cytosol/nucleus (Fig. 1H, I and K) after seawater challenge. In addition, the total JNK content in the mitochondria was also elevated (Fig. 1D and F), whereas that in the cytosol/nucleus (Fig. 1H and J) was decreased, indicating JNK translocation from the cytosol/nucleus to mitochondria during ALI/ARDS.

The protective role of DXM in ALI/ARDS was also investigated. DXM pre-treatment significantly alleviated the high level of phosphorylated JNK (Fig. 1A and C), in both the mitochondria (Fig. 1D and G) and cytosol/nucleus (Fig. 1H and K).

#### Protective effects of the mitoJNK-inhibiting peptide Tat-Sab_KIM1_ against ALI/ARDS

**Figure 2.**
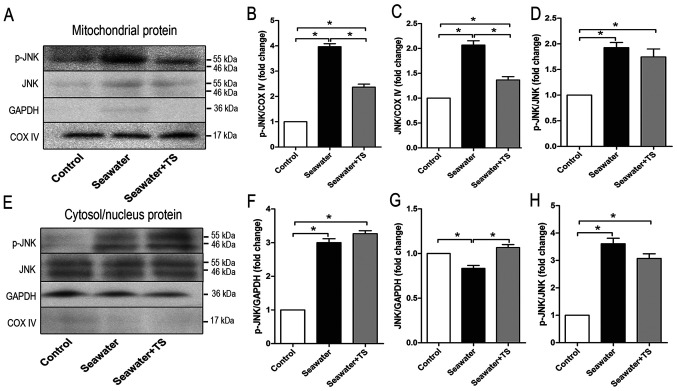
Tat-Sab_KIM1_ peptide tracheal injection could inhibit JNK-mitochondrial activation during acute lung injury/acute respiratory distress syndrome. (A) Representative western blotting images of p-JNK and JNK expression in the mitochondrial protein. Relative (B) p-JNK and (C) JNK protein content in the mitochondrial protein. (D) Normalized ratio of p-JNK/JNK in the mitochondrial protein. (E) Representative western blotting images of p-JNK and JNK expression in the cytosol/nucleus protein. Relative (F) p-JNK and (G) JNK protein content in the cytosol/nucleus protein. (H) Normalized ratio of p-JNK/JNK in the cytosol/nucleus protein. Data are presented as the mean ± SEM. n=4/group. *P<0.05. TS, Tat-Sab_KIM1_; p-, phosphorylated; COX IV, cytochrome c oxidase IV.

To further examine the role of mitoJNK activation in ALI/ARDS, the peptide Tat-Sab_KIM1_, which can act on the Sab_KIM1_ domain and be expressed in mitochondria (6), was used. The peptide can also mediate the localization of JNK by selectively blocking JNK translocation to mitochondria both *in vitro* and *in vivo* (12,26). However, Tat-Sab_KIM1_ does not exhibit any effect on the translocation of JNK to the nucleus (12). Tracheal injection of Tat-Sab_KIM1_ 10 min before modelling significantly decreased the phosphorylation level of JNK in mitochondria (Fig. 2A and B) and total JNK expression in mitochondria when compared with the seawater inhalation group (Fig. 2A and C); however, it did not influence the ratio of p-JNK/JNK when compared with the seawater inhalation group (Fig. 2D). p-JNK expression in the cytosol/nucleus was elevated in both the seawater inhalation group and Tat-Sab_KIM1_ pre-treatment group compared with that in the control group (Fig. 2E and F). The ratio of p-JNK/JNK in the cytosol/nucleus was also elevated after seawater inhalation compared with that in the control group (Fig. 2E and H). In addition, as presented in Fig. 2E and G, Tat-Sab_KIM1_ increased total JNK expression in the cytosol/nucleus when compared with the seawater inhalation group. These results indicated that Tat-Sab_KIM1_ could specifically inhibit JNK localization to the mitochondria and the activation of mitoJNK signaling, without any impact on the ratio of p-JNK/JNK or the cytosolic/nuclear JNK activation.

**Figure 3.**
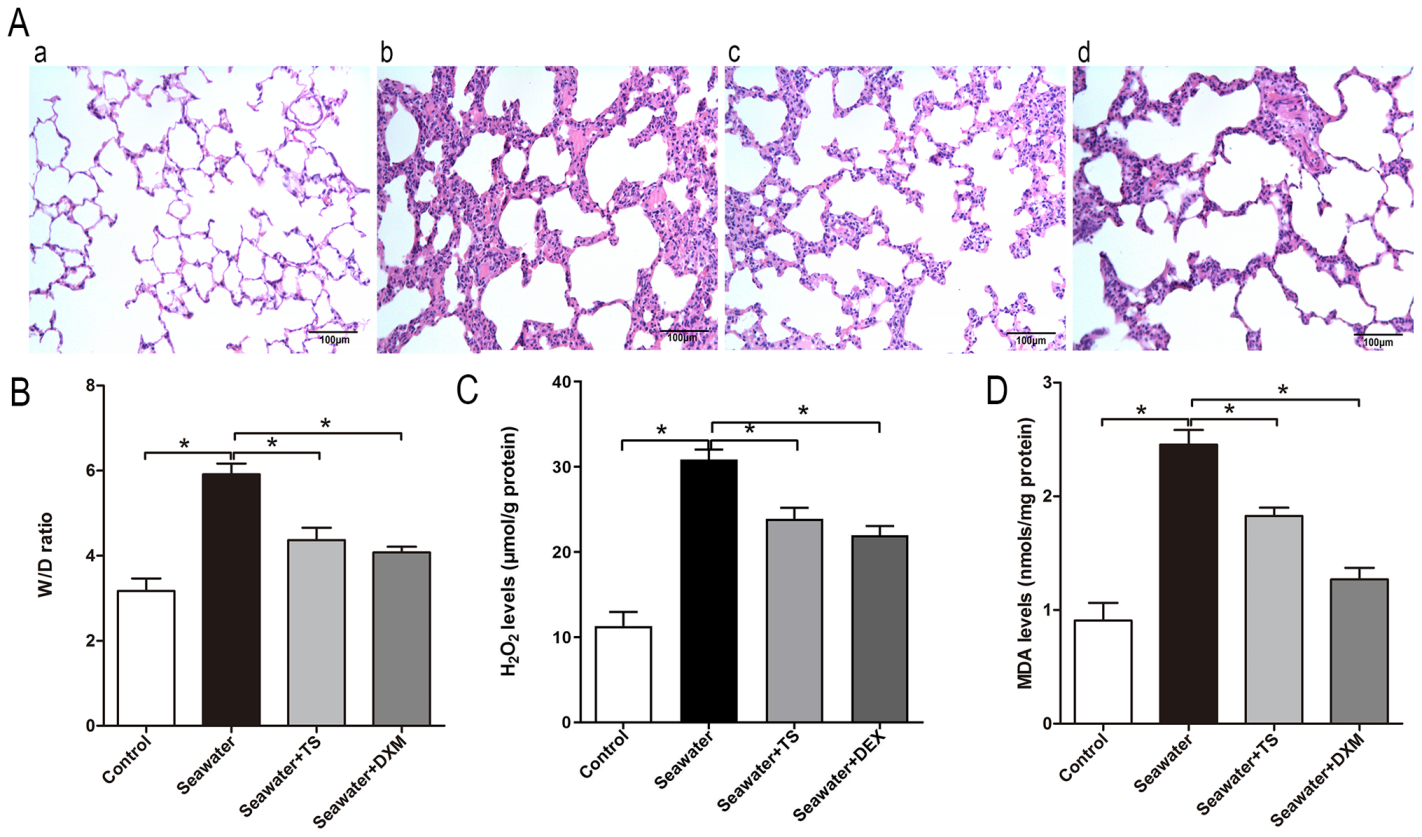
Protective effect of JNK-mitochondrial signaling-inhibiting peptide Tat-Sab_KIM1_ against ALI/ARDS. (A) Histological changes in rat lung tissue; (a) control group, (b) seawater inhalation group, (c) DXM pretreatment group and (d) Tat-Sab_KIM1_ peptide pretreatment group. Scale bar, 100 µm. (B) Seawater inhalation-induced lung W/D ratio changes. Change of (C) H_2_O_2_ and (D) MDA levels. Data are presented as the mean ± SEM. n=4/group. *P<0.05. TS, Tat-Sab_KIM1_; W/D, wet-dry; MDA, malondialdehyde; DXM, dexamethasone.

As Tat-Sab_KIM1_ could selectively inhibit mitoJNK, the effects of mitoJNK activation on the progression of ALI/ARDS were further evaluated. Tat-Sab_KIM1_-mediated inhibition of JNK localization to the mitochondria alleviated the seawater inhalation-induced destruction of lung tissue structure (Fig. 3A) and pulmonary oedema (Fig. 3B). In addition, the inhibitory effects slightly improved the levels of the markers of peroxidation and oxidative stress, H_2_O_2_ and MDA, respectively, compared with the seawater inhalation group (Fig. 3C and D). These results suggested that the inhibition of mitoJNK activation exerted a protective effect against ALI/ARDS. DXM pre-treatment could inhibit JNK phosphorylation in both the mitochondria and cytosol/nucleus compared with that in the seawater group, thus indicating that it did not selectively inhibit mitoJNK (Fig. 1C and G). As shown in Fig. 3A, DXM could also alleviate seawater inhalation-induced lung injury. In addition, the pulmonary oedema and oxidative stress of the DXM pre-treatment group were ameliorated compared with in the seawater inhalation group (Fig. 3B-D).

#### Effects of mitoJNK activation on the occurrence of autophagy

**Figure 4.**
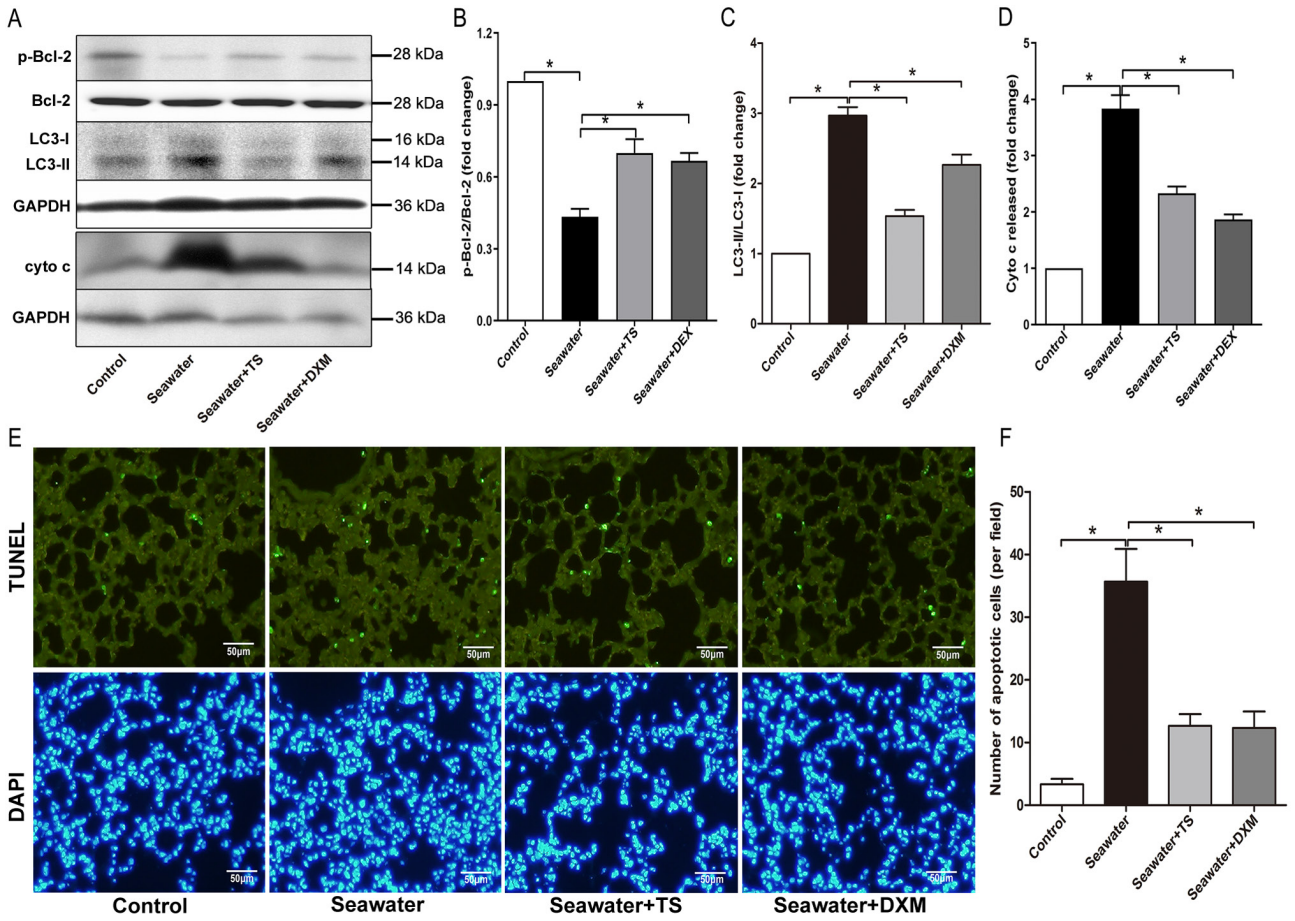
JNK-mitochondrial activation contributes to the occurrence of autophagy and mitochondria-mediated apoptosis. (A) Representative western blotting images of p-Bcl-2, LC3 and cyto c release. (B) Relative p-Bcl-2 protein content. (C) Normalized ratio of LC3-II/LC3-I. (D) Relative content of cyto c released into cytosol. (E) Representative photomicrographs of *in situ* detection of apoptotic cells using TUNEL staining in lung slices from rats. Scale bar, 50 µm. (F) Statistical results of apoptotic cells per field. Data are presented as the mean ± SEM. n=4/group. *P<0.05. TS, Tat-SabKIM1; cyto c, cytochrome c; DXM, dexamethasone.

Autophagy has been reported to participate in the development of ALI/ARDS, as per the conversion of LC3, i.e., LC3-I to LC3-II (27). Bcl-2 may also modulate autophagy via an interaction with the autophagy protein, Beclin 1 (28). Seawater inhalation decreased the expression level of p-Bcl-2 and increased the conversion of LC3 to its active form LC3-II, while the inhibition of mitoJNK by Tat-Sab_KIM1_ restored the expression levels of p-Bcl-2 and LC3 (Fig. 4A-C), thereby indicating that the inhibition blocked the autophagy induced by ALI/ARDS.

#### MitoJNK activation contributes to mitochondria-mediated apoptosis

**Figure 5.**
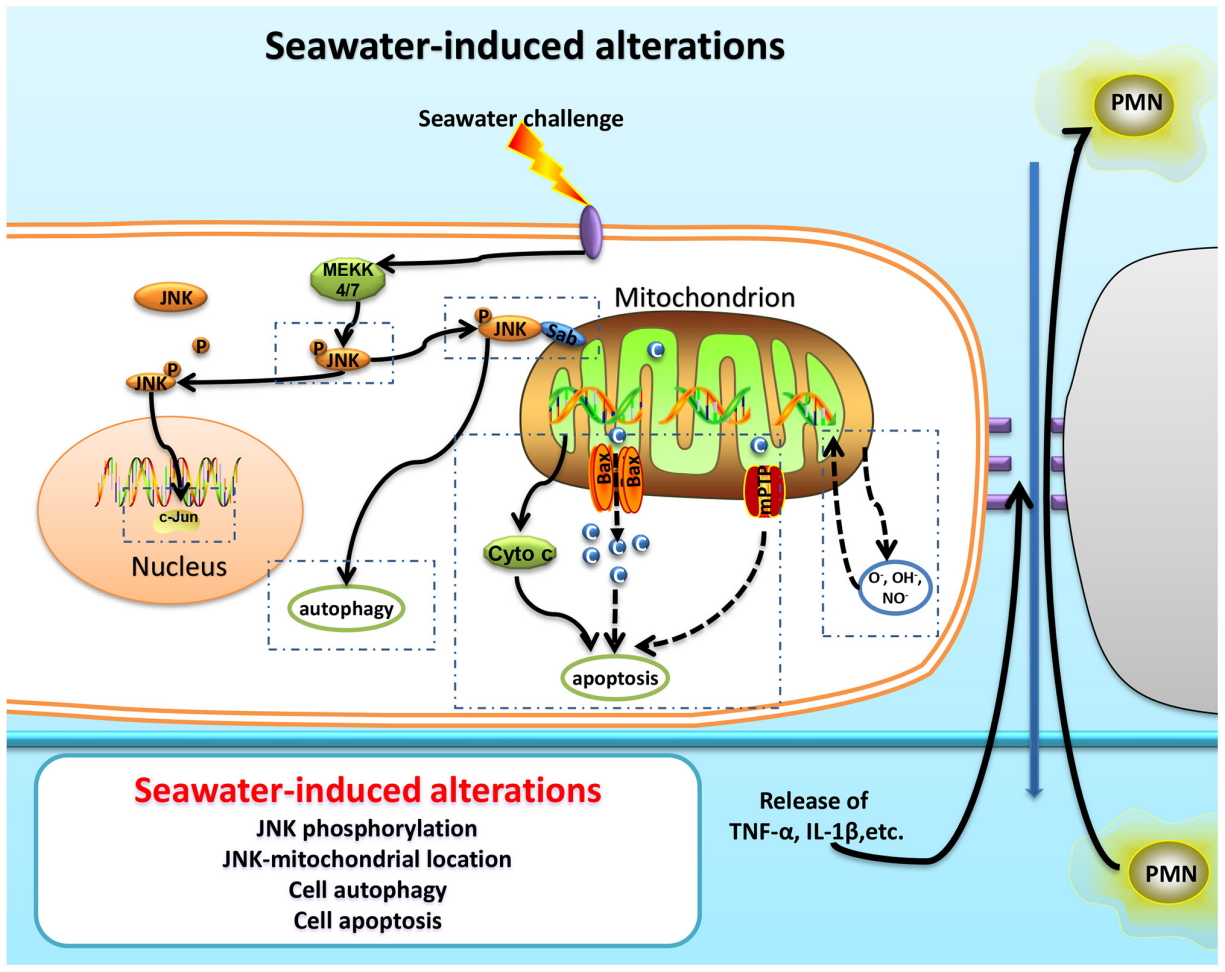
Schematic image depicting the seawater inhalation-induced alterations associated with JNK-mitochondrial activation. PMN, polymorphonuclear cells; C, calcium ion; mPTP, mitochondrial permeability transition pore; Cyto c, cytochrome c; MEKK, MEK kinase; p, phosphorylation.

Next, mitochondria-mediated apoptosis during ALI/ARDS was examined. In the seawater inhalation group, cytochrome c released from mitochondria into the cytosol was significantly increased compared with the control group (Fig. 4A and D). Furthermore, treatment with Tat-Sab_KIM1_ or DXM reduced the amount of cytochrome c released from the mitochondria. The findings observed for cell apoptosis, as evaluated via TUNEL staining, were in line with the aforementioned results. The inhibition of mitoJNK signaling had an anti-apoptotic effect (Fig. 4E and F), as Tat-Sab_KIM1_ treatment significantly decreased the number of TUNEL-positive cells. These results indicated that mitoJNK signaling participated in mitochondria-mediated apoptosis during ALI/ARDS (Fig. 5).

## Discussion

ARDS is one of the leading causes of morbidity and mortality in critically ill patients, and in the USA, ~75,000 individuals die due to ARDS every year (29). Although research and clinical trials have been conducted, the mortality of ARDS continues to be 25–40%, and few pharmacological interventions can improve the mortality rate (30). The pathological changes associated with ALI/ARDS are partly due to the abnormal regulation of mitochondria, including the production of ROS, induced autophagy and apoptosis (17–19). Moreover, maintaining the stability of mitochondrial function is vital to ameliorate ALI/ARDS (20,21).

The main aim of the present study was to confirm that the activation of mitoJNK signaling was involved in the development of ALI/ARDS. By inhibiting the interaction of JNK and Sab using a selective peptide, Tat-Sab_KIM1_, JNK could not be localized to mitochondria, thereby rendering an opportunity to examine the underlying role of mitoJNK in the development of ALI/ARDS. The present results demonstrated that JNK was phosphorylated and activated during ALI/ARDS, not only in the routine JNK pathway but also in the mitoJNK pathway. Furthermore, the mitoJNK signal was activated during seawater inhalation-induced ALI/ARDS, as shown by JNK translocation from the cytosol/nucleus to mitochondria. Tat-Sab_KIM1_ can specifically inhibit JNK localization to mitochondria and the activation of the mitoJNK signaling without any impact on the ratio of p-JNK/JNK or the cytosolic/nuclear JNK activation. Thus, the present study used the Tat-Sab_KIM1_ peptide to inhibit mitoJNK, which demonstrated a protective effect on seawater inhalation-induced ALI/ARDS. In addition, the protective effect was associated with mitoJNK. For example, Bcl-2-regulated autophagy and mitochondria-mediated apoptosis were inhibited by Tat-Sab_KIM1_ pretreatment.

Several studies (31–33) have reported that JNK translocates to the nucleus after its activation and is involved in cellular functions via the phosphorylation of transcription factors in the nucleus. However, some studies (11,34–36) have suggested that JNK not only serves a role in the nucleus, but also in the localization and regulation in other parts of the cells by binding to a specific protein, such as Sab (11). The carboxyl terminus of the Sab protein harbors a kinase interaction motif (KIM), which is similar to c-Jun, and JNK can bind to Sab through this motif (11,37). Moreover, the carboxyl terminus of Sab also harbors four serine/-proline residues, which can also be a site that is phosphorylated by JNK (11). Therefore, Sab can not only interact with JNK but also act as the phosphorylated substrate of JNK (11). Several conformational studies (8,11,26,34,35,37) have reported that Sab was localized to the mitochondria, and that a certain amount of JNK can translocate to the mitochondria by binding to the organelle. The activation of JNK and its interaction with mitochondria are involved in the regulation of mitochondrial functions (12,34). The current experimental results also suggested the occurrence of mitoJNK activation in ALI, which may inhibit the energy production of mitochondria, decrease the mitochondrial membrane potential and lead to mitochondrial dysfunction. Therefore, inhibiting the translocation of JNK to the mitochondria could be used to repair damage by protecting the normal physiological function of the organelle. Previous studies (8,34) have shown that inhibiting Sab expression using gene silencing technology can maintain the normal mitochondrial membrane potential and reduce the apoptosis of cells. Moreover, Chambers *et al* (8,37) revealed that the presence of KIM1 was an essential factor for the binding and interaction between JNK and Sab. The synthesis of the KIM1-specific binding peptide Tat-Sab_KIM1_ may easily and effectively block the interaction between Sab and JNK, thereby inhibiting the localization of JNK to mitochondria (6,37). In addition, previous studies (6,37) demonstrated that Tat-Sab_KIM1_ could successfully reach the cytoplasm via the cell membrane, and its concentration was stable; the concentration after 24 h in the cells could reach up to 90% of the initial concentration (37). Therefore, Tat-Sab_KIM1_ may be used to block the binding of Sab and JNK.

There are some limitations of the present study. Firstly, as ARDS has numerous causes, further studies are required to determine whether Tat-Sab_KIM1_ is effective against numerous causes of ARDS. Secondly, the protective mechanism underlying blocking JNK-mitochondria interaction needs to be fully studied.

In conclusion, as anticipated, during ALI/ARDS, the activation of JNK can disrupt the normal physiological functions of the mitochondria. This disorder leads to the accumulation of ROS and increased cell apoptosis, which is a key event during ALI/ARDS. Through selective inhibition of JNK and Sab binding using Tat-Sab_KIM1_, an effective and stable blocker of JNK-mitochondria interaction, the normal function of mitochondria can be maintained and the deterioration of from ALI/ARDS is blocked.

## Data Availability

The datasets used and/or analyzed during the current study are available from the corresponding author on reasonable request.
